# The Role(s) of Eicosanoids and Exosomes in Human Parturition

**DOI:** 10.3389/fphys.2020.594313

**Published:** 2020-12-23

**Authors:** Eman Mosaad, Hassendrini N. Peiris, Olivia Holland, Isabella Morean Garcia, Murray D. Mitchell

**Affiliations:** ^1^School of Biomedical Science, Institute of Health and Biomedical Innovation – Centre for Children’s Health Research, Faculty of Health, Queensland University of Technology, Brisbane, QLD, Australia; ^2^School of Medical Science, Griffith University, Southport, QLD, Australia

**Keywords:** exosomes, eicosanoids, prostaglandins, pregnancy, parturition, gestation, preterm labor

## Abstract

The roles that eicosanoids play during pregnancy and parturition are crucial to a successful outcome. A better understanding of the regulation of eicosanoid production and the roles played by the various end products during pregnancy and parturition has led to our view that accurate measurements of a panel of those end products has exciting potential as diagnostics and prognostics of preterm labor and delivery. Exosomes and their contents represent an exciting new area for research of movement of key biological factors circulating between tissues and organs akin to a parallel endocrine system but involving key intracellular mediators. Eicosanoids and enzymes regulating their biosynthesis and metabolism as well as regulatory microRNAs have been identified within exosomes. In this review, the regulation of eicosanoid production, abundance and actions during pregnancy will be explored. Additionally, the functional significance of placental exosomes will be discussed.

## Introduction

The fetal membranes perform unique functions to support fetal development and respond to signals for parturition. The correct timing for triggering this process is critical for the successful outcome of the pregnancy. The parturition process is mediated by a combination of signals from the fetus, placenta and mother. There are mainly two signallers of parturition that are interdependent and well reported, namely fetal endocrine signals and fetal growth-related signals (Challis et al., 2005; Menon, 2016; Mesiano, 2019). Both pathways directly and indirectly induce higher production of eicosanoids (particularly prostaglandins) which are important signaling molecules that affect the contractile activity of the myometrium leading to parturition (Challis et al., 2005; Reinl and England, 2015). Hence, administration of specific prostaglandins (E_2_ or F_2α_) is proven to effectively induce labor and cervical ripening (E_2_) in women. Additionally, a better understanding of the regulation of eicosanoid production and the roles played by the various end products during pregnancy and parturition has led to our view that accurate measurements of a panel of those end products has exciting potential as diagnostics and prognostics of preterm labor and delivery (Mitchell et al., 2015).

In this review, we explore the roles and distribution of eicosanoids in the human uterus and fetal membrane during parturition. We also describe exosome abundance during pregnancy and parturition. Finally, we discuss the potentially pivotal role of exosomes in distributing eicosanoids and the related diagnostic and therapeutic potential that this brings.

## Eicosanoids

The term “eicosanoid” has evolved overtime as a definitive term for products of a family of polyunsaturated (C_20_) fatty acids; including, but not limited to, lipoxins, leukotrienes, thromboxanes and prostaglandins. The biosynthesis of eicosanoids and their structural properties are well characterized in mammals (Smith, 1989). Eicosanoids are not stored, and their biosynthesis occurs in all mammalian tissues as a response to hormonal stimulation or mechanical trauma, acting as paracrine or autocrine modulators (Esser-von Bieren, 2017; Strauss and FitzGerald, 2019). Their actions are mediated by the activation of membrane receptors (Kim and Luster, 2007).

### Eicosanoid Biosynthesis

**FIGURE 1:**
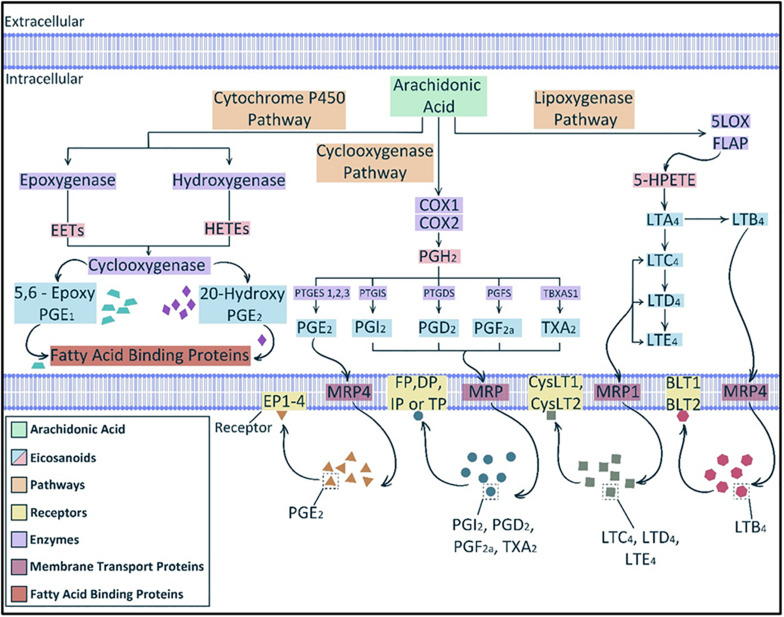
Eicosanoid biosynthesis. The main three pathways involved in eicosanoids biosynthesis from the main precursor, arachidonic acid, are lipoxygenase, cyclooxygenase and cytochrome P450 pathways. The cytochrome P450 pathway produces epoxyeicosatrienoic acids (EETs) and hydroxyeicosatetraenoic acids (HETEs) from which some products can be further metabolized by cyclooxygenase. Cyclooxygenases (COX1 and 2) can act directly on arachidonic acid to produce the unstable intermediate prostaglandin (PG) H_2_ (PGH_2_) which then can produce various prostanoids such as PGE_2_, PGI_2_, PGD_2_, and PGF_2α_ and thromboxane A_2_ (TXA_2_). Lipoxygenase pathway yields leukotrienes (LTs), such as LTA_4_ and LTB_4_. Multidrug resistant proteins (MRPs) facilitate the transfer of eicosanoids through the cell membrane. Multiple cellular membrane receptors mediate the action of eicosanoids, such as EP1-4 for PGE_2_ and BLT1-2 for LTB_4_. [After (Funk, 2001; Strauss and FitzGerald, 2019)].

A first essential and usually rate limiting step in eicosanoid biosynthesis is release of polyunsaturated (C_20_) fatty acids from membrane phospholipid stores (Fitzpatrick and Soberman, 2001). Arachidonic acid is the major common precursor of eicosanoids and its release is precisely regulated by several types of phospholipase A_2_ (Burke and Dennis, 2009) or phospholipase C and subsequent mono- and diacylglycerol lipases (Kano et al., 2009; Hanna and Hafez, 2018). Once released arachidonic acid is converted enzymatically to various eicosanoids via three main pathways (Figure 1): namely, the cyclooxygenase pathway, the lipoxygenase pathway, and the cytochrome P-450 epoxygenase pathway (Strauss and FitzGerald, 2019).

The major products of the cyclooxygenase (COX) pathway are prostanoids such as prostaglandins, thromboxanes and prostacyclin. There are two major COX enzymes that initiate the synthesis of prostaglandins, COX-1 is mainly expressed constitutively, and COX-2 is often induced via cytokines, growth factors and hormones (Herschman, 1996; Smith et al., 1996). Prostaglandin H_2_ (PGH_2_), a direct product of arachidonic acid release and reaction to COX enzymes, is converted to individual prostanoids that are tissue specific by the action of corresponding isomerases and synthases (Smith et al., 2000). For instance, thromboxane A_2_ (TXA_2_) synthase is expressed in platelets and macrophages; prostaglandin I_2_ (PGI_2_), also known as prostacyclin, synthase is expressed in endothelial cells and prostaglandin F_2α_ (PGF_2α_) synthase is abundant in the uterus (Ni et al., 2003; Ueno et al., 2005).

The lipoxygenase pathway produces leukotrienes (LTs). Products of this pathway in leukocytes are part of the LT family of lipid mediators, whose synthesis is mainly initiated by inflammatory cells. Formation of LTs is initiated via hydroperoxyl eicosatetraenoic acid (HPETE) formation from arachidonic acid by 5-lipoxygenase (5-LOX). 5-LOX in turn requires the cooperation of an accessory protein known as five-lipoxygenase activating protein (FLAP). Most HPETE molecules are converted to leukotriene A4 (LTA_4_). LTA_4_ can serve *in vitro* as a precursor for the transcellular biosynthesis of lipoxins and can undergo multiple routes of transformation (Bäck et al., 2011).

The cytochrome P450 epoxygenase pathway produces mainly epoxy-eicosatrienoic acids (EETs) via the catalysis of monooxygenation of arachidonic acid (Smith, 1989; Strauss and FitzGerald, 2019). However, hydroxygenases can also convert arachidonic acid to hydroxy-eicosatetraenoic acids (HETEs) (Strauss and FitzGerald, 2019).

### Transport and Function

Despite the lipid nature of eicosanoids, they do not penetrate the cell membrane freely. Efflux transporters, such as multidrug-resistance proteins (MRPs), are necessary to transport newly synthesized eicosanoids outside the manufacturing cells. Additionally, the cellular uptake of eicosanoids is regulated by organic anion transporter proteins (Funk, 2001). The abundance of eicosanoid receptors and transporters is a limiting factor for their action. Therefore, they are believed to act as local or paracrine effectors initiating specific biochemical reactions in certain tissues.

Due to the different mechanisms that eicosanoids can induce on the cellular level, there are discrete receptor for each compound that mediate its action within the cell (Olson and Ammann, 2007). Thus far, there are 13 distinct cloned and characterized receptors for eicosanoids, including nine for cyclooxygenase-derived prostanoids and four for lipoxygenase-derived leukotrienes (Funk, 2001; Narumiya and Furuyashiki, 2011; Woodward et al., 2011). The nine prostanoid receptors mediate eicosanoid actions via cyclic AMP (cAMP), phosphatidylinositol turnover and Ca^2 +^ shifts (Table 1).

**TABLE 1:** Eicosanoid receptors and their functional properties.

Eicosanoid category	Ligand	Receptor	Functional properties
Cyclooxygenase pathway (prostanoids)	TXA_2_	TP	Increase intracellular calcium, Contractile
	PGI_2_	IP	Increase intracellular cAMP, Relaxing
	PGFzoc	FP	Increase intracellular calcium, Contractile
	PGD_2_	DPI	Increase intracellular cAMP, Relaxing
		DP2	Induce intracellular calcium mobilization and chemoattractant
	PGE_2_	EP1	Increase intracellular calcium, Contractile
		EP3	Inhibit cAMP production, Inhibitory
			Increase intracellular cAMP, Relaxing
Lipoxygenase pathway (leukotrienes)	LTB_4_	BLT1 BLT2	Induce intracellular calcium mobilization and inhibit cAMP production
	LTD_4_	CysLTl	Increase intracellular calcium
	LTC_4_, LTD_4_	CysLT2	Increase intracellular calcium

**BLT1 -2*: Leukotriene B_4_ receptor 1-2; *cAMP*: Cyclic AMP; *CysLT1-2*: Cysteinyl leukotriene receptor 1-2; *DP1-2*: Prostaglandin D_2_ receptor 1-2; *EP1-4*: Prostaglandin E_2_ receptor 1-4; *FP*: Prostaglandin F_2α_ receptor; *IP*: Prostaglandin I_2_ receptor; *LTB*_4_: Leukotriene B_4_; *LTC*_4_: Leukotriene C_4_; *LTD*_4_: Leukotriene D_4_; *PGD*_2_: Prostaglandin D_2_; *PGE*_2_: Prostaglandin E_2_; *PGF_2α_*: Prostaglandin F_2α_; *PGI*_2_: Prostaglandin I_2_; *TP*: Thromboxane receptor; *TXA*_2_: Thromboxane A_2_.*

Despite the short lifespan of eicosanoids, their biological effects are robust. Their biological properties have been studied in many contexts such as the cardiovascular system, immune system, nervous system and gastrointestinal tract as well as in inflammatory settings (Strauss and FitzGerald, 2019). The roles of eicosanoids in reproductive physiology are extensively studied in seminal fluid (Samuelsson, 1963; Alexandre et al., 2007; Remes Lenicov et al., 2012; Szczykutowicz et al., 2019), luteolytic actions (Vijayakumar and Walters, 1983; Bennegård et al., 1991; Miceli et al., 2001) and uterine physiology in pregnancy (Peiris et al., 2017); however, the roles of specific eicosanoids are still being elucidated. In the following sections, we will focus on the role of eicosanoids in uterine physiology during pregnancy and parturition.

### Eicosanoids in Pregnancy and Parturition

The strong relationship between eicosanoids and pregnancy has been recognized for many years. Eicosanoids have various roles in the reproduction process, including ovulation, corpus luteum function, luteolysis, fertilization and decidualisation as well as parturition as previously reviewed (Strauss and FitzGerald, 2019). COX-2-derived PGE_2_ was found to play an important role in oocyte maturation and fertilization by affecting the activity of the cumulus cells surrounding the oocyte (McAdam et al., 1999). Defective embryo implantation and decidualisation were also observed in COX-2-deficient mice uteri, indicating the fundamental role of PGs in normal uterus physiology (Lim et al., 1999; McAdam et al., 1999).

The importance of eicosanoids in parturition has been subjected to detailed investigations using knockdown animal models. For example, parturition defects were observed in rodents deficient of COX enzymes and PGF_2α_ receptor. Mice with targeted disruption of COX-1 gene had delayed parturition, resulting in neonatal death (Gross et al., 1998; Yu et al., 2005). PGF_2α_ receptor-deficient mice, generated by gene knockdown, did not show the normal decline of serum levels of progesterone associated with parturition and consequentially were unable to deliver normal fetuses at term (Sugimoto et al., 1997). Additionally, many clinical observations have accumulated evidences that demonstrate the likely regulatory function of PGs on myometrial contractility and cervical softening. For instance, administration of PGs biosynthesis inhibitors such as aspirin or specific COX-2 (also known as prostaglandin endoperoxide synthase-2; PGHS or PTGS-2) inhibitors extend gestational length, however, does not prevent parturition (Lewis and Schulman, 1973; Collins and Turner, 1975; Khanprakob et al., 2012; Illanes et al., 2014; Triggs et al., 2020). Likewise, administration of PGE_2_ and PGF_2α_ at any stage of gestation leads to increasing uterine contractile activity and cervical ripening (Embrey, 1971). Consequently, PGs are used clinically as a treatment to induce labor (Thomas et al., 2014). Furthermore, production of PGE_2_ and PGF_2α_ increases during late stages of gestation and were found to be associated with the onset of parturition (Romero et al., 1994a, 1996; Slater et al., 1999). This confirms the notion that increased intrauterine PG biosynthesis is a cause rather than a result of the parturition process.

#### Term Labor and Intrauterine Prostaglandin Concentrations

During pregnancy, there are two main groups of regulatory factors that control the contractile activity of the uterus, uterotropins and uterotonins. Uterotropins and relaxatory uterotonins, such as progesterone and PGI, respectively, enhance myometrial relaxation and modulate uterine function and growth (Ilicic et al., 2020). Contrarily, stimulatory uterotonins, such as PGs, can induce contractions of the uteri. Before the parturition process starts, a relaxation state of the myometrium with minimum sensitivity to stimulatory uterotonins, such as PGs, is controlled by progesterone (Mesiano, 2004; Ilicic et al., 2020). Progesterone is a key player in the establishment and maintenance of pregnancy and its role and regulation have been extensively studied in human and experimental models (Arck et al., 2007; Forde et al., 2009; Solano and Arck, 2020). Progesterone withdrawal usually indicates the initiation of the parturition process with changes in the contractile activity of the myometrium. Human parturition is also associated with progesterone receptor subtypes changes (Merlino et al., 2007; Patel et al., 2015).

Although the required enzymes and receptors necessary for the synthesis and action of PGs are present in human myometrial tissue (Astle et al., 2007; Arulkumaran et al., 2012), their concentrations in the uterus may vary during various stages of gestation. During pregnancy, both maternal and fetal tissues produce PGE_2_ and PGF_2α_. The increased intrauterine prostaglandin concentrations are key players in initiating and progressing labor and this occurs before the onset of labor (Romero et al., 1994a, 1996).

During the initial stage of the parturition process, myometrial cellular expression of PG-related genes is significantly increased: these genes include PG biosynthetic enzymes and PG receptors (Challis et al., 2002). The changed expression of these genes in turn increases the uterine tissue sensitivity to the elevated production of PGE_2_ and PGF_2α_. This leads to greater contractile activity that leads to expulsion of the fetus and sequentially expulsion of the placenta (Challis, 2013).

The balance between PG biosynthesis and metabolizing activities in the fetal membranes plays an important role in the parturition process. Intrauterine PG biosynthesis via PGHS occurs in the amnion and to a lesser extent in the chorion, decidua and myometrium. Conversely, prostaglandin dehydrogenase (PGDH) enzyme, which controls the conversion of PGE_2_ and PGF_2α_ to their inactive forms, is predominantly expressed in the chorion before the onset of labor. This leads to the prevention of active amnion-derived PGs reaching the myometrium due to the abundant presence of PGDH in the chorion which lies between the amnion and maternal tissues (Mesiano, 2019).

During parturition, expression of PGHS increases in the chorion, decidua and myometrium. In the meantime, expression of PGDH decreases in the chorion. This leads to greater abundance of active PGs in the chorion and permitting more PGE_2_ and PGF_2α_ to reach and induce their contractile action on the myometrium leading to progression of labor.

Of note, progesterone stimulates PGDH and has been reported to inhibit PTGS2 in the relaxed state of the myometrium before the onset of the parturition process (Pomini et al., 2000; Patel et al., 2003). Conversely, placental cortisol and corticotrophin-releasing hormone (CRH) can stimulate PTGS2 and inhibit PGDH, causing increased access of active PGs to the myometrium (Olson and Ammann, 2007).

PG receptors also play a crucial role in regulating PG action during human parturition. Receptors for PGI_2_, PGE_2_, PGF_2α_ and thromboxane are expressed in the myometrium during pregnancy (Grigsby et al., 2006). PGF_2α_ receptor (FP) and thromboxane receptor (TP) enhance contractions by increasing the intracellular calcium (Ricciotti and FitzGerald, 2011). Both PGI_2_ and PGE_2_ have contrary contractile actions on the myometrium. PGI_2_ receptor (IP) mediates elevated levels of cAMP which in turn leads to relaxation. However, PGI_2_ has been found to play a role in increasing expression of contraction-associated proteins, such as PTGS2 and PG receptors. Interestingly, PGE_2_ has four different receptors (EP1–4) with different physiologic actions. While contractile activity increases when PGE_2_ interacts with EP1 and EP3, relaxation of the tissue can be mediated by PGE_2_ interaction with EP2 and EP4 (Kotani et al., 1995). Therefore, PGE_2_ can cause myometrial contraction or relaxation dependent upon the expression of receptor in different stages of pregnancy and during parturition.

A large literature illustrates the involvement of PGs in the five physiological events of human parturition: fetal membrane rupture via stimulating matrix mettaloproteinase activity and cell apoptosis (McLaren et al., 2000; Keelan et al., 2001), cervical ripening and dilation (Fletcher et al., 1993; Keirse, 1993; Steetskamp et al., 2020), myometrial contractility (Olson and Ammann, 2007), placental separation and uterine involution (Leung et al., 1987). This indicates the importance of further understanding the role of eicosanoids play in prognosis of pregnancy outcomes and their potential role as a diagnostic biomarker for fetus abnormalities and pregnancy complications, such as preeclampsia, gestational diabetes and preterm labor (Dalle Vedove et al., 2016; Hong et al., 2016; Aung et al., 2019; Welch et al., 2020).

#### Preterm Labor and Inflammatory Mechanisms

Labor that occurs before 37 completed weeks of gestation is considered as preterm, and preterm birth is the leading cause of perinatal mortality and morbidity (Goldenberg et al., 2008). The reasons behind the early onset of labor are not clearly identified (Green et al., 2005). Maternal infection is strongly correlated with preterm labor, such as intrauterine infection (Doi, 2020; Romero et al., 1994b). However, preterm delivery is associated with many other risk factors such as multifetal pregnancy, maternal obesity, maternal age, maternal nutrition and socioeconomic status (Johansson et al., 2014; Joseph et al., 2014; Koullali et al., 2016).

Inflammatory mechanisms are significantly involved in term and preterm labor (Christiaens et al., 2008; Peiris et al., 2019, 2020). Many studies focused on identifying labor-associated inflammatory genes profile, such as genes regulating cytokines, chemokines and related factors [reviewed in (Keelan et al., 2003)] which found to be upregulated in term deliveries and more apparently in preterm deliveries (Marvin et al., 2002; Mitchell, 2016). In term labor, infiltration of inflammatory cells increases in the cervix, myometrium, chorioamniotic membranes, and amniotic cavity. This is also found to be associated with increased expression and production of pro-inflammatory cytokines, such as interleukin (IL)-1β, IL-6 and tumor necrosis factor-α (TNFα), and chemokines, such as IL-8 and growth-related oncogene-α (GROα) (Keelan et al., 2003; Romero et al., 2006). Cytokine regulation of intrauterine prostaglandin production was found to be at the biosynthesis level and the catabolic inactivation level. For instance, IL-1β and TNF-α enhance upregulated expression of PGHS leading to increased biosynthesis of prostaglandins by gestational tissues (Hansen et al., 1999; Rauk and Chiao, 2000). Similarly, pro-inflammatory cytokines, IL-1β and TNFα may inhibit PGDH leading to decreased degradation of prostaglandins (Brown et al., 1998; Mitchell et al., 2000). The role of pro-inflammatory cytokines in regulating prostaglandin production is further evidence of the importance of inflammatory mechanisms in mediating parturition (Gross et al., 2000; Peiris et al., 2019, 2020).

Similarly, infection and non-infection-induced inflammation have been found to be associated with preterm labor (Yoon et al., 2001; Romero et al., 2014, 2015). There are many experimental and clinical evidences in support of the involvement of inflammation in preterm labor. For example, pregnant animal models with intrauterine infection or with exposure to microbial products can lead to preterm delivery [reviewed in (Elovitz and Mrinalini, 2004)]. Extrauterine and sub-clinical intrauterine maternal infections have been associated with premature parturition (Gomez et al., 1995; Romero et al., 2006). The production of pro-inflammatory cytokines such as IL-1β, IL-8, and IL-6 are usually increased in the amnion, decidua and myometrium in pregnancies with infection (Goldenberg et al., 2000). This confirms the notion that parturition is a consequence of failure of the maternal immune system to regulate inflammatory mechanisms (Romero et al., 2006).

#### Eicosanoid Distribution and Measurement

Due to the importance of eicosanoids in pregnancy and parturition, the accurate and specific measurement of eicosanoids is critical to our ability to enhance diagnostic and therapeutic strategies for preterm labor. However, the misidentification of PGs has been problematic with traditional methodologies such as immunoassays (Glass et al., 2005). Previously we reviewed the molecular resemblance between eicosanoids and associated compounds that may interfere and affect the specificity of immunoassays (Glass et al., 2005; Peiris et al., 2017). Thus, the gold standard of mass spectrometry that allows full identification of PGs is vital to any meaningful approach to this problem (Peiris et al., 2020).

Prostaglandins are produced by all tissues in the body. Hence measurements of circulating concentrations reflect overall changes in production and cannot be directly linked to a specific tissue or organ source. Moreover, due to rapid clearance of circulating eicosanoids by lungs and kidneys (Golub et al., 1975; Dunn and Hood, 1977; Peiris et al., 2017), we can only assess circulating metabolites of eicosanoids not the original compounds. Therefore, there is a strong argument for evaluating the utility of exosomes (which have a content that reflects the tissue/cellular source) as a stable biomarker for measuring and identifying eicosanoids from specific organs such as the uterus.

## Exosomes

### Exosome Morphology

Exosomes are a subtype of membrane bound extracellular vesicles (EVs); they are 30–120 nm in diameter and have a cup-shaped structure and a lipid bilayer which is similar in orientation of transmembrane constituents to that of the parental cells membrane (Record, 2014; Barile and Vassalli, 2017; Shao et al., 2018). Exosomes contain a diverse array of biologically active molecules such as proteins, lipids, RNA (mRNA, microRNA and noncoding RNA), DNA, protein mediators and eicosanoids (Pillay et al., 2017; Saez et al., 2018). Exosomal contents comprise specific proteins, lipids or genetic materials reflecting the source cell’s physiological state, and can therefore serve as representative biomarkers (Menon et al., 2017).

### Exosome Biogenesis

**FIGURE 2:**
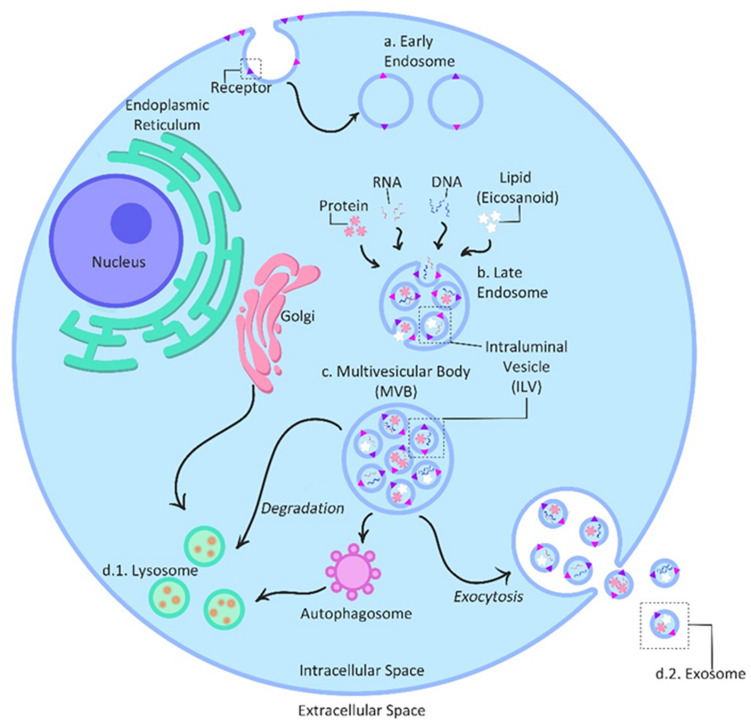
Exosome biogenesis and secretion. The biogenesis of exosomes is initiated with the inward budding of the peripheral membrane into early endosomes **(a)**. Selective exosome packaging with molecules such as protein, RNA and DNA and lipids occurs within the originating cell and cellular compartments such as the Golgi apparatus, endoplasmic reticulum, plasma membrane, nucleus and cytosol. Invagination of the early endosomes into late endosomes **(b)** allows these diverse molecules to be taken up and individually packaged inside intraluminal vesicles (ILVs), turning the endosomes into multivesicular bodies (MVBs) **(c)**. During the invagination process, proteins are incorporated into the membrane, leaving the cytosolic components to be engulfed into the ILVs. Finally, MVBs either release ILVs intracellularly to be absorbed by lysosomes/autophagosomes **(d.1)** for degradation or fuse with the plasma membrane to secrete ILVs out into the extracellular space as exosomes **(d.2)**. [After (Kowal et al., 2014)].

The biogenesis of exosomes involves the inward budding of the peripheral membrane and invagination of the late endosomes also known as Multivesicular Bodies (MVB) (Record, 2014); followed by the formation of intraluminal vesicles (ILVs) inside of the MVB’s (Zhang et al., 2019). During the invagination process, proteins are incorporated into the membrane, leaving the cytosolic components to be engulfed into the ILVs (Zhang et al., 2019). MVBs are then fused with the plasma membrane of the cell releasing ILVs out into the extracellular space; once released ILVs are then referred to as exosomes (Kowal et al., 2014; Zhang et al., 2019; Figure 2). Exosomes biogenesis also requires; Endosomal Sorting Complexes for Transport (ESCRT) 0–III. This complex contains families of vacuolar sorting proteins, other associated proteins (e.g., Alix and tetraspanin) and lipids which also participate in protein sorting and ILV formation during biogenesis (Kowal et al., 2014). The selective packaging of molecules into exosomes occurs within the originating cell (Pillay et al., 2017); constituents within exosomes come from an array of cellular components such as the Golgi apparatus, endoplasmic reticulum, plasma membrane, nucleus and cytosol (Record, 2014), meaning that exosomes can represent many different parts of the cell. The few known selective mechanisms that regulate cargo sorting into exosomes have recently reviewed (Anand et al., 2019).

### Exosome Secretion and Function

The exocytosis of exosomes is an active secretory process (Record, 2014). MVBs move along microtubules toward the cell’s periphery fusing with the plasma membrane and causing the release of exosomes into the extracellular space (Zhang et al., 2019). Connection of the MVB and the microtubule organization center (MTOC) allows the sectorisation of exosome release, restricting the release of exosomes to non-random areas of the cell membrane (Record, 2014). Exosome release is also dependent on the cells and conditions of their surrounding environment (Kowal et al., 2014; Barile and Vassalli, 2017).

Once exosomes are released, they become involved in communication between cells through cargo delivery to the recipient cells. There are three main types signaling modes; autocrine affects the releasing cell, paracrine affects adjacent cells and endocrine is delivered to distal target cells via the circulation.

Exosomes are a device for both transportation and signaling; through their load of bioactive molecules, they have the innate ability to signal from inside a target cell; both from the periphery and intracellular compartments (Record, 2014).

The function of exosomes is to exchange information through the delivery of cargo to distal and adjacent target cells. In doing so, the interaction of target cells with exosomes results in reprogramming of their phenotype and regulation of their function; functions such as migration, proliferation, angiogenesis, translational activity, metabolism, and apoptosis (Ehrlich et al., 2016; Saez et al., 2018). This reprogramming and regulation consequently alters cellular physiology, and in some cases contributing to different pathological states (Pillay et al., 2017).

### Exosomes in Pregnancy

#### Synthesis and Interactions With Surrounding Environment

Exosomes have been identified in the maternal circulation as early as 6 weeks into gestation (Salomon et al., 2014). As gestational age increases, there is an increase in circulating maternal exosome concentration (Pillay et al., 2017); with the increased exosome burden likely related to placental mass and derived primarily from placental mesenchymal stem cells.

First-trimester trophoblast cells act as environmental sensors, and these cells can respond to the changing environment via the synthesis and release of exosomes (Mitchell et al., 2015). For example, an increase of exosome numbers is observed when the *in vitro* environment has a low oxygen tension and is high in D-glucose concentration; these two factors synergistically interact to regulate the bioactivity and release of exosomes originating from first-trimester trophoblast cells.

The effect of environmental factors on the release of exosomes into the maternal circulation via endocrinal communication is dependent on the integrity and stability of exosomes (Ehrlich et al., 2016). For example, increased release of exosomes from trophoblastic cells is seen as a response to challenging environmental conditions (e.g., elevated glucose concentrations and low oxygen tension) which might disrupt the balance of cytokines (Truong et al., 2017). Cytokines being a necessity for healthy implantation, placentation and successful pregnancy outcome (Mitchell et al., 2015).

#### Exchange, Mediatory Roles, and Other Functions

A function of placenta-derived exosomes is to be a mediator in the progression of pregnancy and cell fate. Exosomes are used in cell-to-cell communication between the placenta and maternal organs. This communication has many functions, one of which is the preparatory function of remote tissues for metabolic and placental changes during gestation (Greening et al., 2016; Jin and Menon, 2018).

Basic functions of exosomes in normal uncomplicated pregnancies are promotion of implantation and communication between endometrium and embryo (Jin and Menon, 2018). *In vitro* studies have also revealed the role of exosomes in differential endothelial cell migration and vascular tube formation. Additionally, exosomes have a pivotal immunoregulatory role via the initiation of activated maternal lymphocytes’ local deletion and induction of maternal t-cell apoptosis, which prevents the degradation of invading trophoblastic cells (Greening et al., 2016; Pillay et al., 2017). Placenta-derived exosomes are also found to play a role in viral infection during pregnancy, where trophoblast cells can transfer the necessary capacity of resistance against viral infection to other nonplacental cells via exosomes (Mouillet et al., 2014).

Exosomes regulate all these functions through the transference of their content into target cells. This regulation of activity can occur with either proximal or distal target cells via different interactions; this includes the modification of the extracellular milieu of the target cell, activation of cell membrane receptors, endocytosis by target cells in which the cell contents are released intracellularly and translational activity (e.g., angiogenesis, proliferation, metabolism and apoptosis). Exosomes can then modify the phenotype of these cells through maternal circulation.

The internalization of exosomes is also found to induce the release of pro-inflammatory cytokines (Greening et al., 2016). Exosomes released due to abnormal environmental factors lead to dysfunction of feto-placental endothelium and other various types of endothelial exosomes (Saez et al., 2018).

The involvement of exosomes in the transcellular metabolism of eicosanoids (and enzymes involved in substrate release for eicosanoids) has been described specifically and in terms of lipid mediators in a series of studies. In these studies exosomes from cells contained the full range of phospholipases and many free fatty acids (Subra et al., 2010; Record et al., 2014). The internalization of exosomes was described and the subsequent utilization of exosome cargo in cell metabolism (Subra et al., 2010) as well as involvement of this intercellular trafficking in pathophysiologies (Record et al., 2014). We have recently reviewed this in detail in an invited publication (Peiris et al., 2017).

Various studies have demonstrated the pivotal role of exosomes during human pregnancy and parturition (Sarker et al., 2014; Mitchell et al., 2015; Menon et al., 2017; Jin and Menon, 2018; Sheller-Miller and Menon, 2020). More interestingly, the potential role of exosomes in diagnosis/prognosis of pregnancy complications has gained a lot of attention in the scientific field in the last two decades. For example, preterm labor was one of the main topic that is under investigation (Cantonwine et al., 2016; Menon and Richardson, 2017; von Linsingen et al., 2017). Likewise, studies on gestational diabetes (Powe, 2017; Liu et al., 2018; Saez et al., 2018) and preeclampsia (Pillay et al., 2016; Nielsen et al., 2017; Navajas et al., 2019) showed differential exosomal contents compared to that in uncomplicated pregnancies.

## Final Remarks

The intercellular communication mediated by exosomes has opened new era of research to study biological processes in healthy and pathophysiological conditions. From a clinical perspective, exosomes are mainly used in two applications: as biomarker detection and biologically active carriers. Exosomes are potential candidates as biomarkers detection tool circulating in blood. Enrichment of specific markers can be improved by exosome isolation and cargo identification (Record et al., 2018). Exosomes may transport proteins, lipids and nucleic acids that can be used as diagnostic or prognostic markers for specific clinical conditions. In this respect, several studies identified potential exosomal markers for early detection, diagnosis, and monitoring of cancer patients (Melo et al., 2015; Jalalian et al., 2019; Makler and Asghar, 2020). Similarly, exosomal contents are now gaining much attention in the field of pregnancy complications and fetal abnormalities (Yang et al., 2020). On the other hand, exosomes are now identified as potential platform for enhanced delivery of specific cargo in vivo, which can be biological compounds or therapeutic agents. The methods of loading exosomes with specific cargos of interest have been recently reviewed (Li et al., 2018; Donoso-Quezada et al., 2020; Mitchell et al., 2020). Exosomes have also inspired researchers to use cell-membrane-cloaked nanoparticles, also called synthetic exosome-mimics, as drug delivery platforms (Hu et al., 2011, 2015; Cao et al., 2016). These different applications of exosomes can provide hope to many patients including women with complicated pregnancies.

The relationship and importance of eicosanoids in pregnancy, labor and parturition are well established and have been an area of research for many decades. However, the limitations of immunoassays in the accurate measurement of specific eicosanoids have hampered research. The development of sensitive and specific mass spectrometry-based method to measure individual eicosanoids (e.g., prostaglandins and prostamides) via the monitoring of characteristic mass fragment pairs for each molecule at their distinct retention times has overcome these technical limitations and for the first time allowed accurate measurement of specific eicosanoids (Mitchell et al., 2016). A small but growing number of clinical studies have adopted mass spectrometric evaluations of these compounds, which has led to important new findings in the areas of labor and uterine infection (Maddipati et al., 2014, 2016; Peiris et al., 2020). The identification of the building blocks and enzymes needed for the synthesis of eicosanoids within exosomes is doubly exciting (Subra et al., 2010; Record et al., 2014). Firstly, the evaluation and quantitation of the cargo may provide a real-time snapshot of the cells’ state. Secondly, exploration of the exosomes’ abilities as vesicles of intercellular communication (i.e., to transport and deliver messages between cells) may further our understanding of the parturition process and provide opportunities to reconsider the mechanisms of pregnancy and parturition.

We postulate that together, understanding and quantitating eicosanoid biosynthesis, metabolism and actions in combination with exosome biology will enable the discovery of diagnostic and prognostic biomarkers for many pregnancy complications including preterm labor.

## Author Contributions

MM conceived the idea. EM and IMG prepared figures. EM, HP, OH, IMG, and MM contributed to manuscript writing. All authors contributed to the article and approved the submitted version.

## Conflict of Interest

The authors declare that the research was conducted in the absence of any commercial or financial relationships that could be construed as a potential conflict of interest. The handling editor declared a past co-authorship with one of the authors MM.

## Abbreviations

*5-LOX*: 5-lipoxygenase
*BLT1-2*: Leukotriene B4 receptor 1-2
*cAMP*: Cyclic AMP
*COX*: Cyclooxygenase
*CRH*: Corticotrophin-releasing hormone
*CysLT1-2*: Cysteinyl leukotriene receptor 1-2
*DP1-2*: Prostaglandin D_2_ receptor 1-2
*EETs*: Epoxy-eicosatrienoic acids
*EP1-4*: Prostaglandin E_2_ receptor 1-4
*ESCRT*: Endosomal Sorting Complexes for Transport
*EVs*: Extracellular vesicles
*FLAP*: Five-lipoxygenase activating protein
*FP*: Prostaglandin F_2α_ receptor
*GRO α*: Growth-related oncogene- α
*HETEs*: Hydroxy-eicosatetraenoic acids
*HPETE*: Hydroperoxyl eicosatetraenoic acid
*IL*: Interleukin
*ILVs*: Intraluminal vesicles
*IP*: Prostaglandin I_2_ receptor
*LTA*_4_: Leukotriene A_4_
*LTB*_4_: Leukotriene B_4_
*LTC*_4_: Leukotriene C_4_
*LTD*_4_: Leukotriene D_4_
*LTs*: Leukotrienes
*MRPs*: Multidrug-resistance proteins
*MTOC*: Microtubule organization center
*MVB*: Multivesicular Bodies
*PG*: Prostaglandin
*PGD*_2_: Prostaglandin D_2_
*PGDH*: prostaglandin dehydrogenase
*PGE*_2_: Prostaglandin E_2_
*PGF_2α_*: Prostaglandin F_2α_
*PGH*_2_: Prostaglandin H_2_
*PGHS* = *PTGS-2*: prostaglandin endoperoxide synthase-2
*PGI*_2_: Prostaglandin I_2_
*TNF α*: Tumor necrosis factor- α
*TP*: Thromboxane receptor
*TXA*_2_: Thromboxane A_2_.

## References

[B1] Alexandre B. Lemaire A. Desvaux P. Amar E. (2007). ORIGINAL RESEARCH—ED PHARMACOTHERAPY: intracavernous injections of prostaglandin E1 for erectile dysfunction: patient satisfaction and quality of sex life on long-term treatment. *J. Sex. Med.* 4 426–431. 10.1111/j.1743-6109.2006.00260.x 17367438

[B2] Anand S. Samuel M. Kumar S. Mathivanan S. (2019). Ticket to a bubble ride: cargo sorting into exosomes and extracellular vesicles. *Biochim. Biophys. Acta Proteins Proteom.* 1867:140203. 10.1016/j.bbapap.2019.02.005 30822540

[B3] Arck P. Hansen P. J. Mulac Jericevic B. Piccinni M.-P. Szekeres-Bartho J. (2007). Progesterone during pregnancy: endocrine–immune cross talk in mammalian species and the role of stress. *Am. J. Reprod. Immunol.* 58 268–279. 10.1111/j.1600-0897.2007.00512.x 17681043

[B4] Arulkumaran S. Kandola M. K. Hoffman B. Hanyaloglu A. C. Johnson M. R. Bennett P. R. (2012). The roles of prostaglandin EP 1 and 3 receptors in the control of human myometrial contractility. *J. Clin. Endocrinol. Metab.* 97 489–498. 10.1210/jc.2011-1991 22162473

[B5] Astle S. Newton R. Thornton S. Vatish M. Slater D. M. (2007). Expression and regulation of prostaglandin E synthase isoforms in human myometrium with labour. *Mol. Hum. Reprod.* 13 69–75. 10.1093/molehr/gal093 17105783

[B6] Aung M. T. Yu Y. Ferguson K. K. Cantonwine D. E. Zeng L. McElrath T. F. (2019). Prediction and associations of preterm birth and its subtypes with eicosanoid enzymatic pathways and inflammatory markers. *Sci. Rep.* 9 1–17. 31745121 10.1038/s41598-019-53448-z PMC6863859

[B7] Bäck M. Dahlén S.-E. Drazen J. M. Evans J. F. Serhan C. N. Shimizu T. (2011). International union of basic and clinical pharmacology. LXXXIV: leukotriene receptor nomenclature, distribution, and pathophysiological functions. *Pharmacol. Rev.* 63 539–584. 21771892 10.1124/pr.110.004184

[B8] Barile L. Vassalli G. (2017). Exosomes: therapy delivery tools and biomarkers of diseases. *Pharmacol. Therap.* 174 63–78. 10.1016/j.pharmthera.2017.02.020 28202367

[B9] Bennegård B. Hahlin M. Wennberg E. Norém H. (1991). Local luteolytic effect of prostaglandin F2α in the human corpus luteum. *Fertil. Steril.* 56 1070–1076. 1743324

[B10] Brown N. L. Alvi S. A. Elder M. G. Bennett P. R. Sullivan M. H. (1998). Regulation of prostaglandin production in intact fetal membranes by interleukin-1 and its receptor antagonist. *J. Endocrinol.* 159 519–526. 10.1677/joe.0.1590519 9834469

[B11] Burke J. E. Dennis E. A. (2009). Phospholipase A2 structure/function, mechanism, and signaling. *J. Lipid Res.* 50 S237–S242. 19011112 10.1194/jlr.R800033-JLR200 PMC2674709

[B12] Cantonwine D. E. Zhang Z. Rosenblatt K. Goudy K. S. Doss R. C. Ezrin A. M. (2016). Evaluation of proteomic biomarkers associated with circulating microparticles as an effective means to stratify the risk of spontaneous preterm birth. *Am. J. Obstetr. Gynecol.* 214:631. 10.1016/j.ajog.2016.02.005 PMC4851565 26874302

[B13] Cao H. Dan Z. He X. Zhang Z. Yu H. Yin Q. (2016). Liposomes coated with isolated macrophage membrane can target lung metastasis of breast cancer. *ACS Nano* 10 7738–7748. 27454827 10.1021/acsnano.6b03148

[B14] Challis J. (2013). *Prostaglandins and Parturition. 51st Congress of the German Society for Gynecology and Obstetrics: Gynecology and Gynecological Oncology, Obstetrics, Perinatology and Prenatal Diagnostics, Gynecological Endocrinology and Reproductive Medicine.* Dresden: Springer-Verlag.

[B15] Challis J. R. G. Bloomfield F. H. Bocking A. D. Casciani V. Chisaka H. Connor K. (2005). Fetal signals and parturition. *J. Obstetr. Gynaecol. Res.* 31 492–499. 10.1111/j.1447-0756.2005.00342.x 16343248

[B16] Challis J. R. G. Sloboda D. M. Alfaidy N. Lye S. J. Gibb W. Patel F. A. (2002). Prostaglandins and mechanisms of preterm birth [Review]. *Reproduction* 124 1–17. 10.1530/rep.0.1240001 12090913

[B17] Christiaens I. Zaragoza D. B. Guilbert L. Robertson S. A. Mitchell B. F. Olson D. M. (2008). Inflammatory processes in preterm and term parturition. *J. Reprod. Immunol.* 79 50–57. 18550178 10.1016/j.jri.2008.04.002

[B18] Collins E. Turner G. (1975). Maternal effects of regular salicylate ingestion in pregnancy. *Lancet* 2 335–338. 10.1016/s0140-6736(75)92777-4 51142

[B19] Dalle Vedove F. Fava C. Jiang H. Zanconato G. Quilley J. Brunelli M. (2016). Increased epoxyeicosatrienoic acids and reduced soluble epoxide hydrolase expression in the preeclamptic placenta. *J. Hypertension* 34:1364. 10.1097/HJH.0000000000000942 PMC5559898 27115337

[B20] Doi K. (2020). “Multiple mechanisms of preterm labour other than intrauterine infection,” in *Preterm Labour and Delivery*, ed. Sameshima H. (Singapore: Springer), 89–94.

[B21] Donoso-Quezada J. Ayala-Mar S. González-Valdez J. (2020). State-of-the-art exosome loading and functionalization techniques for enhanced therapeutics: a review. *Crit. Rev. Biotechnol.* 40 804–820. 32605394 10.1080/07388551.2020.1785385

[B22] Dunn M. J. Hood V. L. (1977). Prostaglandins and the kidney. *Am. J. Physiol. Renal Physiol.* 233 F169–F184. 10.1152/ajprenal.1977.233.3.F169 333946

[B23] Ehrlich S. Lambers D. Baccarelli A. Khoury J. Macaluso M. Ho S. M. (2016). Endocrine disruptors: a potential risk factor for gestational diabetes mellitus. *Am. J. Perinatol.* 33 1313–1318. 10.1055/s-0036-1586500 27490770

[B24] Elovitz M. A. Mrinalini C. (2004). Animal models of preterm birth. *Trends Endocrinol. Metab.* 15 479–487. 10.1016/j.tem.2004.10.009 15541647

[B25] Embrey M. (1971). PGE compounds for induction of labour and abortion. *Ann. N. Y. Acad. Sci.* 180 518–523. 10.1111/j.1749-6632.1971.tb53219.x 5286106

[B26] Esser-von Bieren J. (2017). Immune-regulation and-functions of eicosanoid lipid mediators. *Biol. Chem.* 398 1177–1191. 28622139 10.1515/hsz-2017-0146

[B27] Fitzpatrick F. Soberman R. (2001). Regulated formation of eicosanoids. *J. Clin. Invest.* 107 1347–1351. 11390414 10.1172/JCI13241 PMC209329

[B28] Fletcher H. Mitchell S. Simeon D. Frederick J. Brown D. (1993). Intravaginal misoprostol as a cervical ripening agent. *Br. J. Obstet. Gynaecol.* 100 641–644. 8369246 10.1111/j.1471-0528.1993.tb14230.x

[B29] Forde N. Carter F. Fair T. Crowe M. A. Evans A. C. O. Spencer T. E. (2009). Progesterone-regulated changes in endometrial gene expression contribute to advanced conceptus development in cattle1. *Biol. Reprod.* 81 784–794. 10.1095/biolreprod.108.074336 19553605

[B30] Funk C. D. (2001). Prostaglandins and leukotrienes: advances in eicosanoid biology. *Science* 294 1871–1875. 11729303 10.1126/science.294.5548.1871

[B31] Glass M. Hong J. Sato T. A. Mitchell M. D. (2005). Misidentification of prostamides as prostaglandins. *J. Lipid Res.* 46 1364–1368. 15863842 10.1194/jlr.C500006-JLR200

[B32] Goldenberg R. L. Culhane J. F. Iams J. D. Romero R. (2008). Epidemiology and causes of preterm birth. *Lancet* 371 75–84. 18177778 10.1016/S0140-6736(08)60074-4 PMC7134569

[B33] Goldenberg R. L. Hauth J. C. Andrews W. W. (2000). Intrauterine infection and preterm delivery. *N. Engl. J. Med.* 342 1500–1507. 10.1056/nejm200005183422007 10816189

[B34] Golub M. Zia P. Matsuno M. Horton R. (1975). Metabolism of prostaglandins A1 and E1 in man. *J. Clin. Invest.* 56 1404–1410. 1202078 10.1172/JCI108221 PMC333118

[B35] Gomez R. Ghezzi F. Romero R. Muñoz H. Tolosa J. E. Rojas I. (1995). Premature labour and intra-amniotic infection: clinical aspects and role of the cytokines in diagnosis and pathophysiology. *Clin. Perinatol.* 22 281–342. 10.1016/S0095-5108(18)30286-0 7671540

[B36] Green N. S. Damus K. Simpson J. L. Iams J. Reece E. A. Hobel C. J. (2005). Research agenda for preterm birth: recommendations from the March of Dimes. *Am. J. Obstet. Gynecol.* 193(Pt 1) 626–635. 10.1016/j.ajog.2005.02.106 16150253

[B37] Greening D. W. Nguyen H. P. Elgass K. Simpson R. J. Salamonsen L. A. (2016). Human endometrial exosomes contain hormone-specific cargo modulating trophoblast adhesive capacity: insights into endometrial-embryo interactions. *Biol. Reprod.* 94:38. 10.1095/biolreprod.115.134890 26764347

[B38] Grigsby P. L. Sooranna S. R. Adu-Amankwa B. Pitzer B. Brockman D. E. Johnson M. R. (2006). Regional expression of prostaglandin E2 and F2alpha receptors in human myometrium, amnion, and choriodecidua with advancing gestation and labour. *Biol. Reprod.* 75 297–305. 10.1095/biolreprod.106.051987 16707767

[B39] Gross G. Imamura T. Vogt S. K. Wozniak D. F. Nelson D. M. Sadovsky Y. (2000). Inhibition of cyclooxygenase-2 prevents inflammation-mediated preterm labour in the mouse. *Am. J. Physiol. Regul. Integr. Comp. Physiol.* 278 R1415–R1423. 10.1152/ajpregu.2000.278.6.R1415 10848506

[B40] Gross G. A. Imamura T. Luedke C. Vogt S. K. Olson L. M. Nelson D. M. (1998). Opposing actions of prostaglandins and oxytocin determine the onset of murine labour. *Proc. Natl. Acad. Sci. U.S.A.* 95 11875–11879. 10.1073/pnas.95.20.11875 9751758 PMC21733

[B41] Hanna V. S. Hafez E. A. A. (2018). Synopsis of arachidonic acid metabolism: a review. *J. Adv. Res.* 11 23–32. 10.1016/j.jare.2018.03.005 30034873 PMC6052663

[B42] Hansen W. Keelan J. Skinner S. Mitchell M. (1999). Key enzymes of prostaglandin biosynthesis and metabolism. Coordinate regulation of expression by cytokines in gestational tissues: a review. *Prostaglandins Other Lipid Mediat.* 57 243–257. 10402218 10.1016/s0090-6980(99)00008-8

[B43] Herschman H. R. (1996). Prostaglandin synthase 2. *Biochim. Biophys. Acta* 1299 125–140. 10.1016/0005-2760(95)00194-8 8555245

[B44] Hong J.-S. Romero R. Lee D.-C. Than N. G. Yeo L. Chaemsaithong P. (2016). Umbilical cord prostaglandins in term and preterm parturition. *J. Mater. Fetal Neonatal Med.* 29 523–531. 10.3109/14767058.2015.1011120 PMC5769685 25758616

[B45] Hu C.-M. J. Fang R. H. Wang K.-C. Luk B. T. Thamphiwatana S. Dehaini D. (2015). Nanoparticle biointerfacing by platelet membrane cloaking. *Nature* 526 118–121. 26374997 10.1038/nature15373 PMC4871317

[B46] Hu C.-M. J. Zhang L. Aryal S. Cheung C. Fang R. H. Zhang L. (2011). Erythrocyte membrane-camouflaged polymeric nanoparticles as a biomimetic delivery platform. *Proc. Natl. Acad. Sci. U.S.A.* 108 10980–10985. 21690347 10.1073/pnas.1106634108 PMC3131364

[B47] Ilicic M. Zakar T. Paul J. W. (2020). The regulation of uterine function during parturition: an update and recent advances. *Reprod. Sci.* 27 3–28. 32046377 10.1007/s43032-019-00001-y

[B48] Illanes S. E. Perez-Sepulveda A. Rice G. E. Mitchell M. D. (2014). Preterm labour: association between labour physiology, tocolysis and prevention. *Expert Opin. Invest. Drugs* 23 759–771. 10.1517/13543784.2014.905541 24717074

[B49] Jalalian S. H. Ramezani M. Jalalian S. A. Abnous K. Taghdisi S. M. (2019). Exosomes, new biomarkers in early cancer detection. *Anal. Biochem.* 571 1–13. 30776327 10.1016/j.ab.2019.02.013

[B50] Jin J. Menon R. (2018). Placental exosomes: a proxy to understand pregnancy complications. *Am. J. Reprod. Immunol.* 79:e12788. 10.1111/aji.12788 PMC5908735 29193463

[B51] Johansson S. Villamor E. Altman M. Bonamy A. K. Granath F. Cnattingius S. (2014). Maternal overweight and obesity in early pregnancy and risk of infant mortality: a population based cohort study in Sweden. *BMJ* 349:g6572. 10.1136/bmj.g6572 25467170 PMC4252825

[B52] Joseph K. S. Fahey J. Shankardass K. Allen V. M. O’Campo P. Dodds L. (2014). Effects of socioeconomic position and clinical risk factors on spontaneous and iatrogenic preterm birth. *BMC Pregnancy Childbirth* 14:117. 10.1186/1471-2393-14-117 24670050 PMC3987165

[B53] Kano M. Ohno-Shosaku T. Hashimotodani Y. Uchigashima M. Watanabe M. (2009). Endocannabinoid-mediated control of synaptic transmission. *Physiol. Rev.* 89 309–380. 19126760 10.1152/physrev.00019.2008

[B54] Keelan J. A. Blumenstein M. Helliwell R. J. A. Sato T. A. Marvin K. W. Mitchell M. D. (2003). Cytokines, prostaglandins and parturition-a review. *Placenta* 24 S33–S46. 10.1053/plac.2002.0948 12842412

[B55] Keelan J. A. Helliwell R. J. Nijmeijer B. E. Berry E. B. Sato T. A. Marvin K. W. (2001). 15-deoxy-Δ12, 14-prostaglandin J2-induced apoptosis in amnion-like WISH cells. *Prostaglandins Other Lipid Mediat.* 66 265–282. 11785780 10.1016/s0090-6980(01)00164-2

[B56] Keirse M. (1993). Prostaglandins in preinduction cervical ripening. Meta-analysis of worldwide clinical experience. *J. Reprod. Med.* 38 89–100. 8429533

[B57] Khanprakob T. Laopaiboon M. Lumbiganon P. Sangkomkamhang U. S. (2012). Cyclo−oxygenase (COX) inhibitors for preventing preterm labour. *Cochrane Database Syst. Rev.* 10:CD007748. 10.1002/14651858.CD007748.pub2 PMC11403559 23076936

[B58] Kim N. D. Luster A. D. (2007). Regulation of immune cells by eicosanoid receptors. *ScientificWorldJournal* 7 1307–1328. 17767352 10.1100/tsw.2007.181 PMC5900949

[B59] Kotani M. Tanaka I. Ogawa Y. Usui T. Mori K. Ichikawa A. (1995). Molecular cloning and expression of multiple isoforms of human prostaglandin E receptor EP3 subtype generated by alternative messenger RNA splicing: multiple second messenger systems and tissue-specific distributions. *Mol. Pharmacol.* 48 869–879. 7476918

[B60] Koullali B. Oudijk M. A. Nijman T. A. Mol B. W. Pajkrt E. (2016). Risk assessment and management to prevent preterm birth. *Semin. Fetal Neonatal Med.* 21 80–88. 10.1016/j.siny.2016.01.005 26906339

[B61] Kowal J. Tkach M. Théry C. (2014). Biogenesis and secretion of exosomes. *Curr. Opin. Cell Biol.* 29 116–125. 10.1016/j.ceb.2014.05.004 24959705

[B62] Leung A. Kwok P. Chang A. (1987). Association between prostaglandin E2 and placental abruption. *Br. J. Obstetr. Gynaecol.* 94 1001–1002. 10.1111/j.1471-0528.1987.tb02279.x 3479999

[B63] Lewis R. B. Schulman J. D. (1973). Influence of acetylsalicylic acid, an inhibitor of prostaglandin synthesis, on the duration of human gestation and labour. *Lancet* 2 1159–1161. 10.1016/s0140-6736(73)92934-6 4127544

[B64] Li S.-P. Lin Z.-X. Jiang X.-Y. Yu X.-Y. (2018). Exosomal cargo-loading and synthetic exosome-mimics as potential therapeutic tools. *Acta Pharmacol. Sin.* 39 542–551. 29417947 10.1038/aps.2017.178 PMC5888690

[B65] Lim H. Gupta R. A. Ma W. G. Paria B. C. Moller D. E. Morrow J. D. (1999). Cyclo-oxygenase-2-derived prostacyclin mediates embryo implantation in the mouse via PPARdelta. *Genes Dev.* 13 1561–1574. 10.1101/gad.13.12.1561 10385625 PMC316805

[B66] Liu J. Wang S. Wang Q. Du J. Wang B. (2018). Gestational diabetes mellitus is associated with changes in the concentration and bioactivity of placental exosomes in the maternal circulation across gestation. *Eur. Rev. Med. Pharmacol. Sci.* 22 2036–2043. 29687860 10.26355/eurrev_201804_14733

[B67] Maddipati K. R. Romero R. Chaiworapongsa T. Chaemsaithong P. Zhou S. L. Xu Z. (2016). Lipidomic analysis of patients with microbial invasion of the amniotic cavity reveals up−regulation of leukotriene B4. *FASEB J.* 30 3296–3307. 27312808 10.1096/fj.201600583R PMC5024690

[B68] Maddipati K. R. Romero R. Chaiworapongsa T. Zhou S. L. Xu Z. Tarca A. L. (2014). Eicosanomic profiling reveals dominance of the epoxygenase pathway in human amniotic fluid at term in spontaneous labour. *FASEB J.* 28 4835–4846. 25059230 10.1096/fj.14-254383 PMC4200329

[B69] Makler A. Asghar W. (2020). Exosomal biomarkers for cancer diagnosis and patient monitoring. *Expert Rev. Mol. Diagn.* 20 387–400. 32067543 10.1080/14737159.2020.1731308 PMC7071954

[B70] Marvin K. Keelan J. Eykholt R. Sato T. Mitchell M. (2002). Use of cDNA arrays to generate differential expression profiles for inflammatory genes in human gestational membranes delivered at term and preterm. *Mol. Hum. Reprod.* 8 399–408. 11912289 10.1093/molehr/8.4.399

[B71] McAdam B. F. Catella-Lawson F. Mardini I. A. Kapoor S. Lawson J. A. FitzGerald G. A. (1999). Systemic biosynthesis of prostacyclin by cyclooxygenase (COX)-2: the human pharmacology of a selective inhibitor of COX-2. *Proc. Natl. Acad. Sci. U.S.A.* 96 272–277. 10.1073/pnas.96.1.272 9874808 PMC15129

[B72] McLaren J. Taylor D. Bell S. (2000). Prostaglandin E2-dependent production of latent matrix metalloproteinase-9 in cultures of human fetal membranes. *Mol. Hum. Reprod.* 6 1033–1040. 11044467 10.1093/molehr/6.11.1033

[B73] Melo S. A. Luecke L. B. Kahlert C. Fernandez A. F. Gammon S. T. Kaye J. (2015). Glypican-1 identifies cancer exosomes and detects early pancreatic cancer. *Nature* 523 177–182. 26106858 10.1038/nature14581 PMC4825698

[B74] Menon R. (2016). Human fetal membranes at term: dead tissue or signalers of parturition? *Placenta* 44 1–5. 10.1016/j.placenta.2016.05.013 27452431 PMC5375105

[B75] Menon R. Mesiano S. Taylor R. N. (2017). Programmed fetal membrane senescence and exosome-mediated signaling: a mechanism associated with timing of human parturition. *Front. Endocrinol.* 8:196. 10.3389/fendo.2017.00196 28861041 PMC5562683

[B76] Menon R. Richardson L. S. (2017). Preterm prelabour rupture of the membranes: a disease of the fetal membranes. *Semin. Perinatol.* 41 409–419. 28807394 10.1053/j.semperi.2017.07.012 PMC5659934

[B77] Merlino A. A. Welsh T. N. Tan H. Yi L. J. Cannon V. Mercer B. M. (2007). Nuclear progesterone receptors in the human pregnancy myometrium: evidence that parturition involves functional progesterone withdrawal mediated by increased expression of progesterone receptor-A. *J. Clin. Endocrinol. Metab.* 92 1927–1933. 17341556 10.1210/jc.2007-0077

[B78] Mesiano S. (2004). Myometrial progesterone responsiveness and the control of human parturition. *J. Soc. Gynecol. Invest.* 11 193–202. 10.1016/j.jsgi.2003.12.004 15120691

[B79] Mesiano S. (2019). “Chapter 11 – endocrinology of human pregnancy and fetal-placental neuroendocrine development,” in *Yen and Jaffe’s Reproductive Endocrinology (Eighth Edition)*, eds Strauss J. F. Barbieri R. L. (Amsterdam: Elsevier), 256–284e9. 10.1016/B978-0-323-47912-7.00011-1

[B80] Miceli F. Minici F. Pardo M. G. Navarra P. Proto C. Mancuso S. (2001). Endothelins enhance prostaglandin (PGE2 and PGF2α) biosynthesis and release by human luteal cells: evidence of a new paracrine/autocrine regulation of luteal function. *J. Clin. Endocrinol. Metab.* 86 811–817. 10.1210/jcem.86.2.7236 11158051

[B81] Mitchell C. M. (2016). *Transcriptional and Epigenetic Regulation of Labour Associated Inflammatory Genes in the Amnion Faculty of Health.* Callaghan, NSW: The University of Newcastle.

[B82] Mitchell M. D. Crookenden M. A. Vaswani K. Roche J. R. Peiris H. N. (2020). The frontiers of biomedical science and its application to animal science in addressing the major challenges facing Australasian dairy farming. *Anim. Prod. Sci.* 60 1–9. 10.1071/AN18579

[B83] Mitchell M. D. Goodwin V. Mesnage S. Keelan J. A. (2000). Cytokine-induced coordinate expression of enzymes of prostaglandin biosynthesis and metabolism: 15-hydroxyprostaglandin dehydrogenase. *Prostaglandins Leukot Essent Fatty Acids* 62 1–5. 10.1054/plef.1999.0117 10765972

[B84] Mitchell M. D. Peiris H. N. Kobayashi M. Koh Y. Q. Duncombe G. Illanes S. E. (2015). Placental exosomes in normal and complicated pregnancy. *Am. J. Obstetr. Gynecol.* 213 S173–S181. 10.1016/j.ajog.2015.07.001 26428497

[B85] Mitchell M. D. Rice G. E. Vaswani K. Kvaskoff D. Peiris H. N. (2016). Differential regulation of eicosanoid and endocannabinoid production by inflammatory mediators in human choriodecidua. *PLoS One* 11:e0148306. 10.1371/journal.pone.0148306 26840435 PMC4740432

[B86] Mouillet J.-F. Ouyang Y. Bayer A. Coyne C. B. Sadovsky Y. (2014). The role of trophoblastic microRNAs in placental viral infection. *Int. J. Dev. Biol.* 58 281. 10.1387/ijdb.130349ys PMC4377297 25023694

[B87] Narumiya S. Furuyashiki T. (2011). Fever, inflammation, pain and beyond: prostanoid receptor research during these 25 years. *FASEB J.* 25 813–818. 10.1096/fj.11-0302ufm 21357250

[B88] Navajas R. Corrales F. J. Paradela A. (2019). Serum exosome isolation by size-exclusion chromatography for the discovery and validation of preeclampsia-associated biomarkers. *Methods Mol. Biol.* 1959 39–50. 30852814 10.1007/978-1-4939-9164-8_3

[B89] Ni H. Sun T. Ma X.-H. Yang Z.-M. (2003). Expression and regulation of cytosolic prostaglandin E synthase in mouse uterus during the peri-implantation period. *Biol. Reprod.* 68 744–750. 12604621 10.1095/biolreprod.102.007328

[B90] Nielsen M. R. Frederiksen-Møller B. Zachar R. Jørgensen J. S. Hansen M. R. Ydegaard R. (2017). Urine exosomes from healthy and hypertensive pregnancies display elevated level of α-subunit and cleaved α-and γ-subunits of the epithelial sodium channel—ENaC. *Pflügers Arch.* 469 1107–1119. 28405801 10.1007/s00424-017-1977-z

[B91] Olson D. M. Ammann C. (2007). Role of the prostaglandins in labour and prostaglandin receptor inhibitors in the prevention of preterm labour. *Front. Biosci.* 12:1329–1343. 10.2741/2151 17127385

[B92] Patel B. Elguero S. Thakore S. Dahoud W. Bedaiwy M. Mesiano S. (2015). Role of nuclear progesterone receptor isoforms in uterine pathophysiology. *Hum. Reprod. Update* 21 155–173. 25406186 10.1093/humupd/dmu056 PMC4366574

[B93] Patel F. A. Funder J. W. Challis J. R. (2003). Mechanism of cortisol/progesterone antagonism in the regulation of 15-hydroxyprostaglandin dehydrogenase activity and messenger ribonucleic acid levels in human chorion and placental trophoblast cells at term. *J. Clin. Endocrinol. Metab.* 88 2922–2933. 10.1210/jc.2002-021710 12788907

[B94] Peiris H. N. Romero R. Vaswani K. Reed S. Gomez-Lopez N. Tarca A. L. (2019). Preterm labour is characterized by a high abundance of amniotic fluid prostaglandins in patients with intra-amniotic infection or sterile intra-amniotic inflammation. *J. Matern. Fetal Neonatal Med.* 10.1080/14767058.2019.1702953 [Epub ahead of print]. 31885290 PMC8314747

[B95] Peiris H. N. Vaswani K. Almughlliq F. Koh Y. Q. Mitchell M. D. (2017). Review: eicosanoids in preterm labour and delivery: Potential roles of exosomes in eicosanoid functions. *Placenta* 54 95–103. 10.1016/j.placenta.2016.12.013 27988062

[B96] Peiris H. N. Vaswani K. Holland O. Koh Y. Q. Almughlliq F. B. Reed S. (2020). Altered productions of prostaglandins and prostamides by human amnion in response to infectious and inflammatory stimuli identified by mutliplex mass spectrometry. *Prostaglandins Leukot. Essential Fatty Acids* 154:102059. 10.1016/j.plefa.2020.102059 32014738

[B97] Pillay P. Maharaj N. Moodley J. Mackraj I. (2016). Placental exosomes and pre-eclampsia: maternal circulating levels in normal pregnancies and, early and late onset pre-eclamptic pregnancies. *Placenta* 46 18–25. 27697217 10.1016/j.placenta.2016.08.078

[B98] Pillay P. Moodley K. Moodley J. Mackraj I. (2017). Placenta-derived exosomes: potential biomarkers of preeclampsia. *Int. J. Nanomed.* 12 8009–8023. 10.2147/IJN.S142732 29184401 PMC5673050

[B99] Pomini F. Patel F. A. Mancuso S. Challis J. R. (2000). Activity and expression of 15-hydroxyprostaglandin dehydrogenase in cultured chorionic trophoblast and villous trophoblast cells and in chorionic explants at term with and without spontaneous labour. *Am. J. Obstet. Gynecol.* 182(Pt 1) 221–226. 10.1016/s0002-9378(00)70516-3 10649182

[B100] Powe C. E. (2017). Early pregnancy biochemical predictors of gestational diabetes mellitus. *Curr. Diabetes Rep.* 17:12. 10.1007/s11892-017-0834-y 28229385

[B101] Rauk P. N. Chiao J. P. (2000). Interleukin−1 stimulates human uterine prostaglandin production through induction of cyclooxygenase−2 expression. *Am. J. Reprod. Immunol.* 43 152–159. 10735591 10.1111/j.8755-8920.2000.430304.x

[B102] Record M. (2014). Intercellular communication by exosomes in placenta: a possible role in cell fusion? *Placenta* 35 297–302. 10.1016/j.placenta.2014.02.009 24661568

[B103] Record M. Carayon K. Poirot M. Silvente-Poirot S. (2014). Exosomes as new vesicular lipid transporters involved in cell-cell communication and various pathophysiologies. *Biochim. Biophys. Acta* 1841 108–120. 10.1016/j.bbalip.2013.10.004 24140720

[B104] Record M. Silvente-Poirot S. Poirot M. Wakelam M. J. (2018). Extracellular vesicles: lipids as key components of their biogenesis and functions. *J. Lipid Res.* 59 1316–1324. 29764923 10.1194/jlr.E086173 PMC6071772

[B105] Reinl E. L. England S. K. (2015). Fetal-to-maternal signaling to initiate parturition. *J. Clin. Invest.* 125 2569–2571. 26098207 10.1172/JCI82576 PMC4563697

[B106] Remes Lenicov F. Rodriguez Rodrigues C. Sabatté J. Cabrini M. Jancic C. Ostrowski M. (2012). Semen promotes the differentiation of tolerogenic dendritic cells. *J. Immunol.* 189:4777. 10.4049/jimmunol.1202089 23066152

[B107] Ricciotti E. FitzGerald G. A. (2011). Prostaglandins and inflammation. *Arterioscler. Thromb. Vasc. Biol.* 31 986–1000. 21508345 10.1161/ATVBAHA.110.207449 PMC3081099

[B108] Romero R. Espinoza J. Gonçalves L. F. Kusanovic J. P. Friel L. A. Nien J. K. (2006). Inflammation in preterm and term labour and delivery. *Semin. Fetal Neonatal Med.* 11 317–326. 10.1016/j.siny.2006.05.001 16839830 PMC8315239

[B109] Romero R. Gonzalez R. Baumann P. Behnke E. Rittenhouse L. Barberio D. (1994a). Topographic differences in amniotic fluid concentrations of prostanoids in women in spontaneous labour at term. *Prostaglandins Leukot. Essential Fatty Acids* 50 97–104. 10.1016/0952-3278(94)90154-6 8171074

[B110] Romero R. Miranda J. Chaemsaithong P. Chaiworapongsa T. Kusanovic J. P. Dong Z. (2015). Sterile and microbial-associated intra-amniotic inflammation in preterm prelabour rupture of membranes. *J. Matern. Fetal Neonatal Med.* 28 1394–1409. 10.3109/14767058.2014.958463 25190175 PMC5371030

[B111] Romero R. Miranda J. Chaiworapongsa T. Korzeniewski S. J. Chaemsaithong P. Gotsch F. (2014). Prevalence and clinical significance of sterile intra-amniotic inflammation in patients with preterm labour and intact membranes. *Am. J. Reprod. Immunol.* 72 458–474. 10.1111/aji.12296 25078709 PMC4192099

[B112] Romero R. Munoz H. Gomez R. Galasso M. Sherer D. M. Cotton D. (1994b). Does infection cause premature labour and delivery? *Semin. Reprod. Endocrinol.* 17 12–19.

[B113] Romero R. Munoz H. Gomez R. Parra M. Polanco M. Valverde V. (1996). Increase in prostaglandin bioavailability precedes the onset of human parturition. *Prostaglandins Leukot. Essential Fatty Acids* 54 187–191. 10.1016/s0952-3278(96)90015-0 8860106

[B114] Saez T. de Vos P. Sobrevia L. Faas M. M. (2018). Is there a role for exosomes in foetoplacental endothelial dysfunction in gestational diabetes mellitus? *Placenta* 61 48–54. 10.1016/j.placenta.2017.11.007 29277271

[B115] Salomon C. Torres M. J. Kobayashi M. Scholz-Romero K. Sobrevia L. Dobierzewska A. (2014). A gestational profile of placental exosomes in maternal plasma and their effects on endothelial cell migration. *PLoS One* 9:e98667. 10.1371/journal.pone.0098667 24905832 PMC4048215

[B116] Samuelsson B. (1963). Isolation and identification of prostaglandins from human seminal plasma 18. Prostaglandins and related factors. *J. Biol. Chem.* 238 3229–3234. 14085366

[B117] Sarker S. Scholz-Romero K. Perez A. Illanes S. E. Mitchell M. D. Rice G. E. (2014). Placenta-derived exosomes continuously increase in maternal circulation over the first trimester of pregnancy. *J. Transl. Med.* 12:204. 10.1186/1479-5876-12-204 25104112 PMC4283151

[B118] Shao H. Im H. Castro C. M. Breakefield X. Weissleder R. Lee H. (2018). New technologies for analysis of extracellular vesicles. *Chem. Rev.* 118 1917–1950. 10.1021/acs.chemrev.7b00534 29384376 PMC6029891

[B119] Sheller-Miller S. Menon R. (2020). Isolation and characterization of human amniotic fluid-derived exosomes. *Methods Enzymol.* 645 181–194. 10.1016/bs.mie.2020.07.006 PMC12266933 33565971

[B120] Slater D. Dennes W. Sawdy R. Allport V. Bennett P. (1999). Expression of cyclo-oxygenase types-1 and -2 in human fetal membranes throughout pregnancy. *J. Mol. Endocrinol.* 22 125–130. 10.1677/jme.0.0220125 10194515

[B121] Smith W. L. (1989). The eicosanoids and their biochemical mechanisms of action. *Biochem. J.* 259 315–324. 10.1042/bj2590315 2655580 PMC1138513

[B122] Smith W. L. DeWitt D. L. Garavito R. M. (2000). Cyclooxygenases: structural, cellular, and molecular biology. *Annu. Rev. Biochem.* 69 145–182. 10966456 10.1146/annurev.biochem.69.1.145

[B123] Smith W. L. Garavito R. M. DeWitt D. L. (1996). Prostaglandin endoperoxide H synthases (cyclooxygenases)-1 and -2. *J. Biol. Chem.* 271 33157–33160. 10.1074/jbc.271.52.33157 8969167

[B124] Solano M. E. Arck P. C. (2020). Steroids, pregnancy and fetal development [Review]. *Front. Immunol.* 10:3017. 10.3389/fimmu.2019.03017 32038609 PMC6987319

[B125] Steetskamp J. Bachmann E. Hasenburg A. Battista M. J. (2020). Safety of misoprostol for near-term and term induction in small-for-gestational-age pregnancies compared to dinoprostone and primary cesarean section: results of a retrospective cohort study. *Arch. Gynecol. Obstet.* 302 1369–1374. 32761274 10.1007/s00404-020-05703-2

[B126] Strauss J. F. FitzGerald G. A. (2019). “Chapter 4 – steroid hormones and other lipid molecules involved in human reproduction,” in *Yen and Jaffe’s Reproductive Endocrinology (Eighth Edition)*, eds Strauss J. F. Barbieri R. L. (Amsterdam: Elsevier), 75–114e117. 10.1016/B978-0-323-47912-7.00004-4

[B127] Subra C. Grand D. Laulagnier K. Stella A. Lambeau G. Paillasse M. (2010). Exosomes account for vesicle-mediated transcellular transport of activatable phospholipases and prostaglandins. *J. Lipid Res.* 51 2105–2120. 20424270 10.1194/jlr.M003657 PMC2903822

[B128] Sugimoto Y. Yamasaki A. Segi E. Tsuboi K. Aze Y. Nishimura T. (1997). Failure of parturition in mice lacking the prostaglandin F receptor. *Science* 277 681–683. 10.1126/science.277.5326.681 9235889

[B129] Szczykutowicz J. Kałuża A. Kaźmierowska-Niemczuk M. Ferens-Sieczkowska M. (2019). The potential role of seminal plasma in the fertilization outcomes. *BioMed Res. Int.* 2019:5397804. 10.1155/2019/5397804 31531356 PMC6720062

[B130] Thomas J. Fairclough A. Kavanagh J. Kelly A. J. (2014). Vaginal prostaglandin (PGE2 and PGF2a) for induction of labour at term. *Cochrane Database Syst. Rev.* 2014:Cd003101. 10.1002/14651858.CD003101.pub3 24941907 PMC7138281

[B131] Triggs T. Kumar S. Mitchell M. (2020). Experimental drugs for the inhibition of preterm labour. *Expert Opin. Invest. Drugs* 29 507–523. 10.1080/13543784.2020.1752661 32290715

[B132] Truong G. Guanzon D. Kinhal V. Elfeky O. Lai A. Longo S. (2017). Oxygen tension regulates the miRNA profile and bioactivity of exosomes released from extravillous trophoblast cells–liquid biopsies for monitoring complications of pregnancy. *PLoS One* 12:e0174514. 10.1371/journal.pone.0174514 28350871 PMC5370130

[B133] Ueno N. Takegoshi Y. Kamei D. Kudo I. Murakami M. (2005). Coupling between cyclooxygenases and terminal prostanoid synthases. *Biochem. Biophys. Res. Commun.* 338 70–76. 10.1016/j.bbrc.2005.08.152 16140261

[B134] Vijayakumar R. Walters W. A. (1983). Human luteal tissue prostaglandins, 17β-estradiol, and progesterone in relation to the growth and senescence of the corpus luteum. *Fertil. Steril.* 39 298–303. 6572151 10.1016/s0015-0282(16)46875-5

[B135] von Linsingen R. Bicalho M. D. G. de Carvalho N. S. (2017). Baby born too soon: an overview and the impact beyond the infection. *J. Matern. Fetal Neonatal Med.* 30 1238–1242. 10.1080/14767058.2016.1209653 27380453

[B136] Welch B. M. Keil A. P. van ‘t Erve T. J. Deterding L. J. Williams J. G. Lih F. B. (2020). Longitudinal profiles of plasma eicosanoids during pregnancy and size for gestational age at delivery: a nested case-control study. *PLoS Med.* 17:e1003271. 10.1371/journal.pmed.1003271 32797061 PMC7428021

[B137] Woodward D. F. Jones R. L. Narumiya S. (2011). International union of basic and clinical pharmacology. LXXXIII: classification of prostanoid receptors, updating 15 years of progress. *Pharmacol. Rev.* 63 471–538. 10.1124/pr.110.003517 21752876

[B138] Yang H. Ma Q. Wang Y. Tang Z. (2020). Clinical application of exosomes and circulating microRNAs in the diagnosis of pregnancy complications and foetal abnormalities. *J. Transl. Med.* 18:32. 10.1186/s12967-020-02227-w 31969163 PMC6975063

[B139] Yoon B. H. Romero R. Moon J. B. Shim S. S. Kim M. Kim G. (2001). Clinical significance of intra-amniotic inflammation in patients with preterm labour and intact membranes. *Am. J. Obstet. Gynecol.* 185 1130–1136. 10.1067/mob.2001.117680 11717646

[B140] Yu Y. Cheng Y. Fan J. Chen X.-S. Klein-Szanto A. Fitzgerald G. A. (2005). Differential impact of prostaglandin H synthase 1 knockdown on platelets and parturition. *J. Clin. Invest.* 115 986–995. 10.1172/JCI23683 15776109 PMC1064983

[B141] Zhang Y. Liu Y. Liu H. Tang W. H. (2019). Exosomes: biogenesis, biologic function and clinical potential. *Cell Biosci.* 9:19. 10.1186/s13578-019-0282-2 30815248 PMC6377728

